# Monitoring Steam Penetration in Channeled Instruments: An Evidence-Based Worst-Case for Practical Situations

**DOI:** 10.3389/fmedt.2020.566143

**Published:** 2020-11-16

**Authors:** Francesco Tessarolo, Michela Masè, Andrea Visonà, Josephus P. C. M. van Doornmalen Gomez Hoyos

**Affiliations:** ^1^Department of Industrial Engineering, University of Trento, Trento, Italy; ^2^Healthcare Research and Innovation Program (Innovazione e Ricerca in Clinica e Sanità - Health Technology Assessment), Bruno Kessler Foundation, Trento, Italy; ^3^Institute of Mountain Emergency Medicine, Eurac Research, Bolzano, Italy; ^4^STEELCO S.p.A., Treviso, Italy; ^5^Department of Applied Physics, Eindhoven University of Technology, Eindhoven, Netherlands; ^6^Catharina Hospital, Eindhoven, Netherlands

**Keywords:** sterilization, steam penetration, channeled instrument, medical device reprocessing, process monitoring

## Abstract

Steam sterilization of channeled medical devices requires steam penetration into narrow channels. However, a quantitative characterization of this phenomenon in practical situations is lacking. This study evaluates the effect of load, loading pattern, and wrapping system on steam penetration into channels. We tested the hypothesis that a 70 cm tube with one closed end could be representative of the worst case for steam penetration in wrapped channeled instruments in practical conditions. A validated sterilization process was run in a sterilizer equipped with infrared sensors for the measurement of water vapor fraction (WVF). WVF values collected at the closed end of an unwrapped 70 cm reference tube were compared to those obtained at the closed end of wrapped 50 cm test tubes, representative for channeled devices in the clinical practice. The open ends of the test tubes were placed inside packs, testing the effects of different combinations of wrappings, load amounts, and pack positions. The worst case for steam penetration was experimentally defined as the condition showing the lowest WVF value during the exposure phase. WVF values at the closed end of 50 cm long tubes were affected by load amount, wrapping, and pack position. Steam penetration was higher for heavier loads in rigid containers, but lower for heavier loads in soft wrappings (pouch, non-woven fabric, and crepe). In all the tested combinations of load/wrappings related to the clinical practice the 70 cm reference tube displayed lower WVF values than the wrapped 50 cm test tubes, indicating worse steam penetration in the reference than test tubes. Our findings provide experimental evidence that a 70 cm is the worst case in all practical combinations of load and wrapping encountered in the field. The 70 cm tube is a representative for a wrapped 50 cm channel with one end closed and for a wrapped 100 cm channel with both ends open. A measuring system integrating the WVF sensor on a 70 cm tube may provide a physics-based, quantitative steam penetration test for real-time monitoring of the steam sterilization process of channeled instruments.

## Introduction

Steam sterilization is recommended as the preferred sterilization method for medical instruments in hospitals (1–4). Effective surface steam sterilization requires that the saturated steam is able to reach all the surfaces to be sterilized for a defined time interval and temperature (5). Standards for steam sterilization aim at guaranteeing that these conditions are met in any sterilizer and in the presence of any load (6–8). In last decades the development of minimally invasive surgery techniques has led to an increased use of reusable instruments with narrow channels, which need sterilization before use. To properly sterilize channeled instruments, steam penetration into the channels is required, which represents a further challenge for steam sterilization. Despite a large technological effort toward the development of new systems and standards to assess steam penetration into channels, the available tools remain limited to devices using chemical and biological indicators, which do not allow quantitative and operator-independent evaluations (9–11). The development of quantitative methods, based on physical principles, is thus advocated for a reliable characterization of steam penetration into channeled instruments (12).

Recent experimental and modeling studies have helped to clarify the physical processes and the quantitative features underling steam penetration into channels. These studies have pointed out that channels with a constant radius are the most difficult to be steam penetrated (11) and that steam penetration depends on the number of open ends (i.e., one or two) and length of the channels (13–16). Specifically, in channels with two open ends the most difficult location to reach is the center of the inner channel wall, while in channels with only one open end it is the inner channel wall at the closed end (17). Thus, in terms of steam penetration, a channel with two open ends can be represented as a half-length channel with a single open end. As concerns channel length, experimental data, and simulation results reported in the literature have shown that steam penetration is more difficult in longer tubes (18, 19). Channeled instruments with two open ends requiring sterilization in the clinical practice have usually a maximal length of 1 m, which corresponds to a length of 50 cm in channels with a single open end. Therefore, in practical conditions, the most difficult location for steam penetration in a mixed load of channeled and non-channeled instruments is represented by the inner wall of a 50 cm tube with a single end open. Recent works have also pointed out that the internal diameter of the channels used in surgical instruments does not play a major role in steam penetration (11, 13).

Based on these concepts, a new measuring device (4D IR sensor, Miele Group, Gütersloh, Germany) has been recently developed to test steam penetration in channeled instruments. The device is composed of an infrared (IR) steam penetration sensor, which measures the steam fraction at the closed end of a tube with a single open end. The length of the sensor tube can be modified to be representative of different channeled instruments. In particular, the “worst case” condition can be realized by extending the tube length beyond 50 cm. In other words, the device is based on the idea that a 4D IR sensor, equipped with a sufficiently long tube, may provide the reference worst case for steam penetration in any practical situation. In the real setting of hospital sterilization services, the result of a steam sterilization process is determined by several variables (20), which includes the sterilizer, the process, the load and loading pattern, and the microbiological barrier used. While the sterilizer is usually configured by the manufacturer and the process is generally a fixed, programmed cycle, installed on the sterilizer, the load, loading pattern, and microbiological barrier may change in every process run, affecting steam penetration and making each steam sterilization process a unique event. The availability of an evidence-based monitoring system, able to provide quantitative and real-time information about steam penetration in the worst condition for channeled instruments, may help to guarantee adequate steam penetration for any load of instruments processed in the sterilizer. This however is conditioned to a proper choice of the tube length, which needs to be based on experimental evidence acquired under a variety of practically encountered situations, including various wrapping materials, loads, and load locations.

In this perspective, through a well-designed set of experiments, the present study aimed at assessing the effects of the load, loading pattern, and wrapping material on steam penetration into channels. More specifically, we aimed at testing the hypothesis that a tube with 70 cm length (hereafter called “reference” tube) could be representative of the most difficult condition for steam penetration in loads of channeled instruments in practical situations. To this aim, steam fractions were measured and compared at the closed end of the reference tube and of test tubes representative of clinical instruments in a variety of sterilization conditions. Representative test tubes were designed with a 50 cm length (i.e., maximal length of clinically available devices) and their open ends were inserted into packs to mimic the most difficult conditions for steam penetration in the clinical practice. In particular, the adopted configuration required the passage of the steam through the wrapping/packaging system before entering the test tube, as it occurs for steam sterilization of packed channeled instruments in the field. To consider the wide spectrum of conditions encountered in clinical sterilization processes, different combinations of wrapping and load amounts were tested, as well as pack positions in the sterilizer chamber.

## Methods

### Study Design

**Figure 1:**
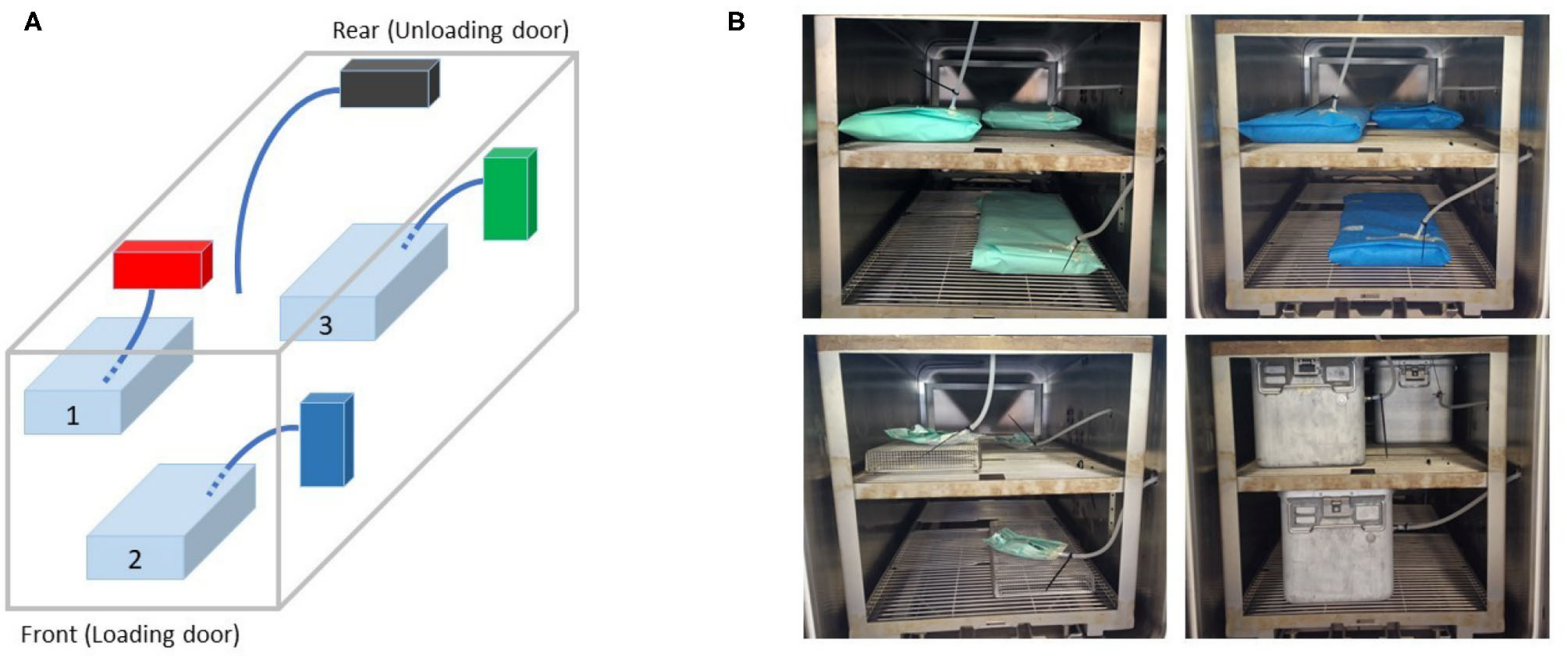
**(A)** Schematic representation of the positions of the packs (shown in light blue with numbered positions), open end tubes (in blue) and 4D infrared (IR) sensors (colored boxes) in the experimental sterilizer chamber (outer gray box). The steam fraction at the closed end of the 70 cm reference tube was measured by the black 4D IR sensor. The 50 cm test tubes had open ends inserted in packs and the steam fraction at their closed ends was measured by the other 4D IR sensors (shown in red, upper front; green, upper rear; blue, bottom front). **(B)** Representative loading patterns for a routine test with crepe (top left), spunbond-meltblown-spunbond (SMS) (top right), pouches (bottom left), and containers (bottom right).

The study was designed as a comparative evaluation of steam fraction values measured by a 4D IR sensor, mounted on a 70 cm stainless-steel tube (reference tube), with respect to three additional equivalent sensors mounted on 50 cm tubes (test tubes). The reference tube had an internal/external (I/E) diameter of 4/6 mm. The test tubes were composed by a sequence of three connected segments: a 10 cm stainless-steel tube (I/E diameter of 4/6 mm) mounted on the sensor, a 30 cm silicone tube (I/E diameter of 5/9 mm), and a 10 cm tube extension in polytetrafluoroethylene (PTFE) (I/E diameter of 5/7 mm). The open end of the reference tube was located at a predefined position in the sterilizer (right side of the unloading door, see Figure 1A). The open ends of the test tubes were inserted in packs to mimic channeled devices in practical situations. The use of a segmented tube enabled a rapid positioning of the packs during the experiments.

To cover the wide spectrum of load configurations encountered in the field, a total of 36 practical conditions were reproduced, which combined the following three variables: type of wrapping, load amount in the pack, and pack position inside the sterilizer chamber. Experiments were performed in quintuplicate for each test condition.

### 4D IR Sensors

Four identical 4D IR sensors (same model and manufacturer) were used in the study. These sensors provide a quantitative measurement of the water vapor fraction (WVF), which is defined as the ratio of the measured water vapor to the maximum water vapor amount at a specific temperature and is expressed as a dimensionless percentage value. All sensors were previously calibrated and gave equivalent WVF measurements when mounting a 70 cm tube.

**Figure 2:**
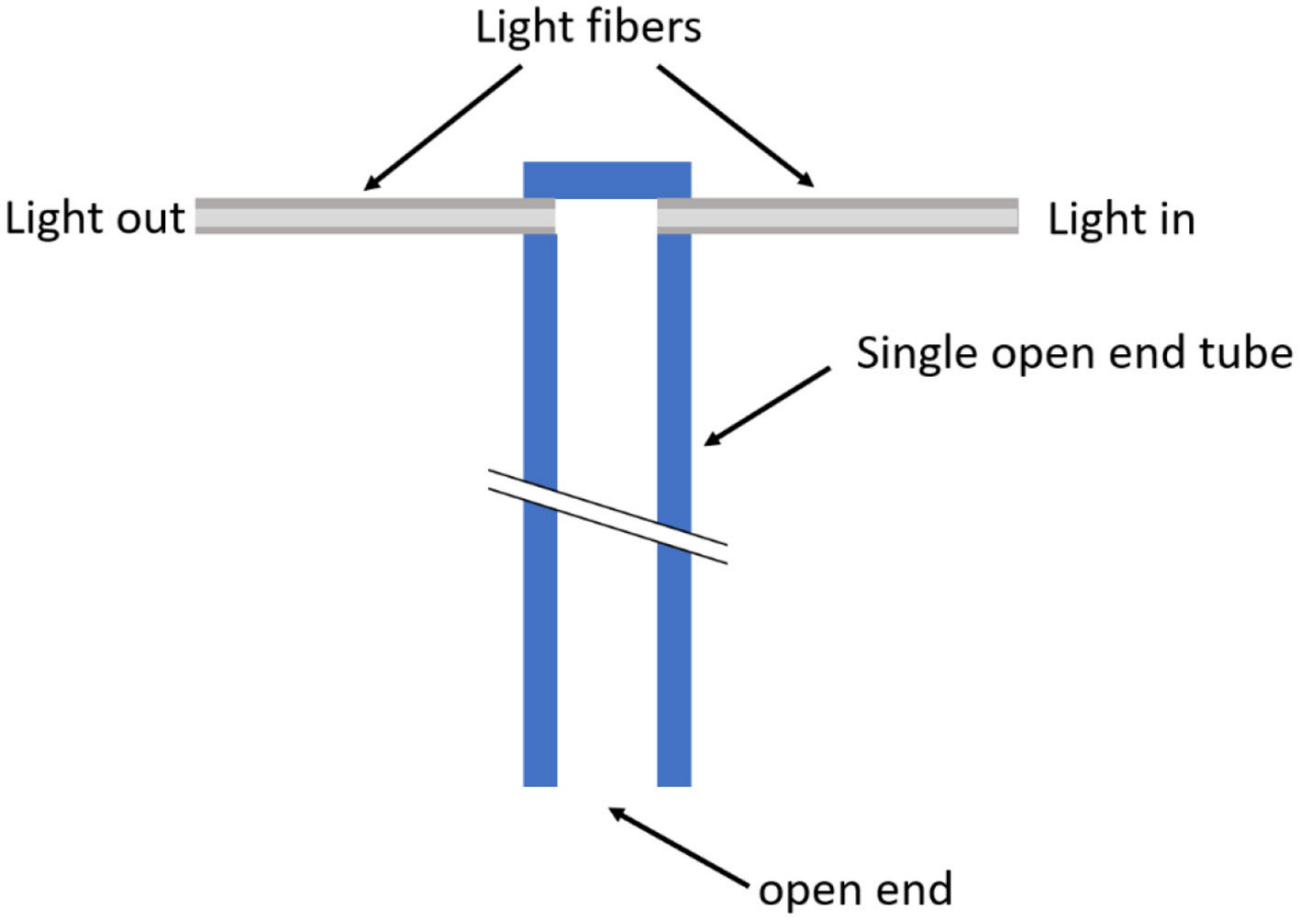
Schematic representation of the sensing unit in a 4D infrared (IR) sensor positioned at the end of a test tube (in blue). The open end of the tube allows steam penetration toward its closed end, where an IR light beam is emitted into the tube via a glass fiber. At the opposite side of the tube a second fiber receives the light transmitted through the gas mixture inside the tube. Adapted with permission from (13).

The working principle and validation process of the sensor has been previously detailed (13). Briefly, each sensor is composed of a tube, a sensing unit, and a control unit. The sensing unit contains the sensor head with two optical fibers a light emitting diode (LED) IR source, and a photodiode for measuring the transmitted light. The IR light is emitted into the inner space of the tube through an optical fiber. At the opposite side, the second optical fiber collects the light transmitted through the gas into the tube (Figure 2). The LED emits light at two different wavelengths. The first wavelength is efficiently absorbed by water molecules and transmitted by non-condensable gases (NCGs), so that the second optical fiber collects less IR light in the presence of a higher number of water molecules. The second wavelength is not significantly affected by the presence of water molecules, and its intensity variation is used to suppress light scattering effects within the tube and/or variations in the whole optical system. Steam fraction values expressed in terms of WVF are obtained by combining light intensity measurements as previously described (13).

The sensor head was mounted outside the sterilizer chamber via a tri-clamp connector fixed to a suitable hose passing through the sterilizer jacket. The control unit hosts the printed circuit board (PCB) and the controlling processor unit, which converts the analogic signals (voltage values proportional to the transmitted light intensities) from the photodiode to digital WVF signals. WVF signals were acquired at a sampling frequency of 2 Hz.

### Steam Sterilizer and Sterilization Process

Experiments were performed in an eight unit VS08PCD (Steelco S.p.a., Riese Pio X, Italy) steam sterilizer, equipped with an internal water degassing system and a boiler. The dimensions of the sterilizer chamber were 670 mm (width) × 700 mm (height) × 1,310 mm (depth), resulting in a total volume of 614 l and usable volume of 609 l. At the beginning of each experimental day, a warming up cycle was run to warm the sterilizer chamber. Then, an air leakage test and a Bowie & Dick (B&D) test were performed in accordance with EN 285 requirements (6) and to ISO 17665-1 recommendations (7) for routine monitoring and control of the sterilization process. The B&D test was performed placing an ETS (3M Sterilization) in the empty sterilizer chamber. Experiments were performed provided that the B&D test was passed [i.e., the sterilizer and the process satisfied the requirements for steam penetration according to the standard ISO 11140-4 (21)]. This protocol granted stable initial conditions for the sterilizer and comparability of experimental results through different experimental sessions.

**Figure 3:**
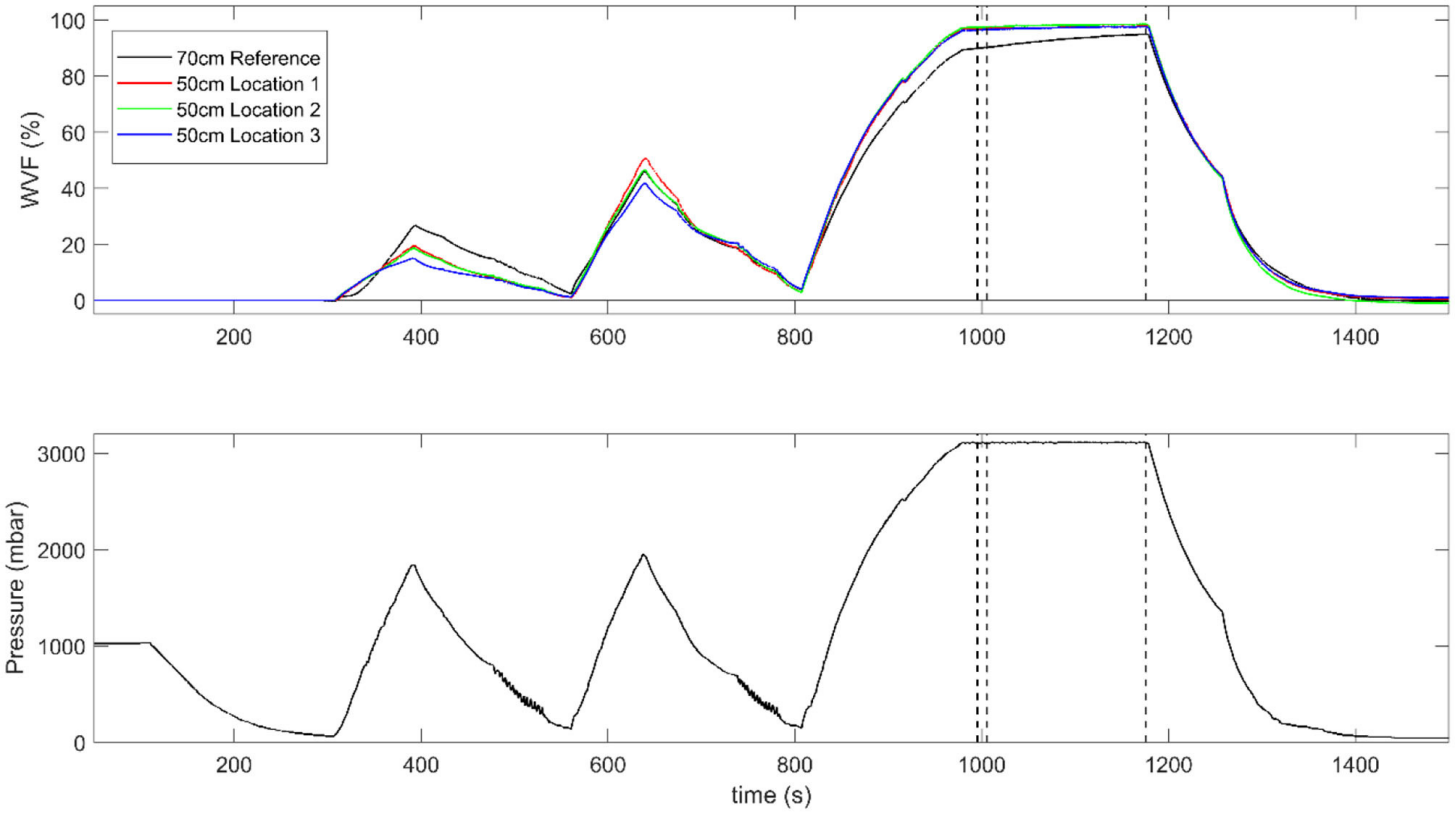
Time course of the steam fraction **(top)** and the sterilizer chamber pressure **(bottom)** during a test sterilization cycle. The steam fraction is expressed in terms water vapor fraction (WVF) and is measured at the closed end of the 50 cm test tubes (red, green, and blue curves) and of the 70 cm reference tube (black curve). In this representative example, measurements were collected in a session where crepe was used as wrapping material and no additional load was present in the packs. The outer dashed lines indicate the start and end of the exposure phase, while the intermediate line marks the end of the 10 s period where WVF differences were calculated.

Experimental sessions were run using the sterilization process cycle shown in Figure 3. The cycle was composed of: (1) evacuation phase to set the chamber pressure to 50 mbar; (2) pre-conditioning phase with two trans-atmospheric pulses (steam injection control point at ~1,900 mbar and vacuum control point at 130 mbar); (3) equilibration phase of 30 s duration to stabilize temperature and pressure in the sterilization range; (4) exposure phase of 180 s duration; (5) drying phase, shortened to reduce experimental time without affecting the sterilization process.

### Wrapping, Load, and Pack Position Into the Sterilizer Chamber

The test tubes were inserted in packs, which were realized using four types of wrappings and three amounts of load, resulting in a total of 12 wrapping/load combinations, as detailed in Table 1.

**Table 1:** Load-wrapping combinations tested in the study.

**Wrapping**	**Description**	**Load amount**		
		**None (kg)**	**Medium (kg)**	**Full (kg)**
Crepe	double wrapping realized with two single foils of medical crepe paper (1,200 × 1,200 mm) (Sogeva, Ospitaletto, Italy)	0	7.5	15
SMS	single wrapping realized using double layered Spunbond-Meltblown-Spunbond (SMS) polypropylene non-woven fabric (QUICK CHECK, H400), O&M Halyard, Inc. Alpharetta, GE, US)	0	7.5	15
Pouch	double wrapping realized using double flat gusseted pouches made of medical paper and polymeric laminate (150 mm × 50 mm × 300 mm) (Sogeva, Ospitaletto, Italy)	0	0.2	0.4
Container	AESCULAP full size, 8 inch high (@30 l volume), with uncolored aluminum lid mounting hydrophobic paper filters (Aesculap AG, Tuttlingen, Germany)	0	7.5	15

*Each combination was used to realize packs placed in three different locations inside the sterilizer chamber*.

A plastic net, loaded with stainless-steel screws of predefined amounts, was used for containers, crepe, and spunbond-meltblown-spunbond (SMS) polypropylene non-woven fabric. An envelope folding was used for realizing the packs with crepe and SMS. Specifically, a sequential double wrapping was used for crepe and a single envelope for double layered SMS. A scalpel incision was performed at the top of the pack and the 10 cm PTFE tube was passed through the sterile barrier system. An air-tight connection between the PTFE tube and the packaging was obtained by sealing adhesive tape around the tube entrance.

In the packs with pouches, the screws were inserted in a pouch, whose open side was sealed by adhesive tape, enabling the 10 cm PTFE tube to pass through. A second pouch was added using similar sealing and pass-through system for the PTFE tube.

Paper filters were positioned on the container slots and changed before each process. A dedicated air-tight pass-through system was mounted on each container to enable the insertion of the 10 cm PTFE tube through the lateral wall of the container. Typical pack configurations are shown in Figure 1B.

Each load/wrapping configuration was tested at three different locations within the sterilizer chamber (upper front level, upper rear level, and lower front level), as shown in Figure 1A. Each location was monitored by the same sensor throughout the experimental sessions.

### Data Acquisition and Analysis

Steam fraction values at the closed end of the test and reference tubes were measured during each sterilization cycle and expressed as WVF percentages.

The quantitative analysis of WVF values was focused on the exposure phase. In this phase, characterized by almost constant temperature and pressure values, steam penetration into the channels is mostly driven by diffusion of water molecules toward the closed end of the tubes according to concentration gradients (22). Considering that diffusion requires time to favor steam penetration (13), the beginning of the exposure phase represents the most critical time to guarantee steam sterilization conditions. A representative value of steam fraction at the beginning of the exposure phase was thus calculated by averaging WVF values over the first 10 s of the exposure phase.

To experimentally test whether the 70 cm reference tube could represent the worst case for steam penetration in channeled instruments, the difference (ΔWVF) between the WVF values of the test tubes and the reference tube was computed at the beginning of the exposure phase for each experimental condition. The mean value and standard error of ΔWVF values for each load/wrapping combination at the three chamber positions were calculated over five replicates. A positive ΔWVF value indicated that the reference tube was the worst case for steam penetration in that experimental condition (i.e., a higher amount of vapor penetrated in the test tubes than in the reference tube).

## Results

A representative plot of steam fraction measurements for the test and reference tubes is reported in Figure 3. As expected, steam penetration was clearly driven by chamber pressure (lower panel) and the WVF reached higher values during the exposure phase (top panel). Differences in steam penetration between test and reference tubes were present during the whole process, although they were particularly evident during the first injection of steam and they progressively decreased during the process.

The comparison of the measurements performed in different experimental conditions showed that steam fraction values in the test tubes were mainly affected by the load/wrapping configurations, although differences were also present among the three pack locations. Differences among positions were larger at the beginning of the conditioning phase, likely due to gas inhomogeneities inside the sterilizer chamber.

**Figure 4:**
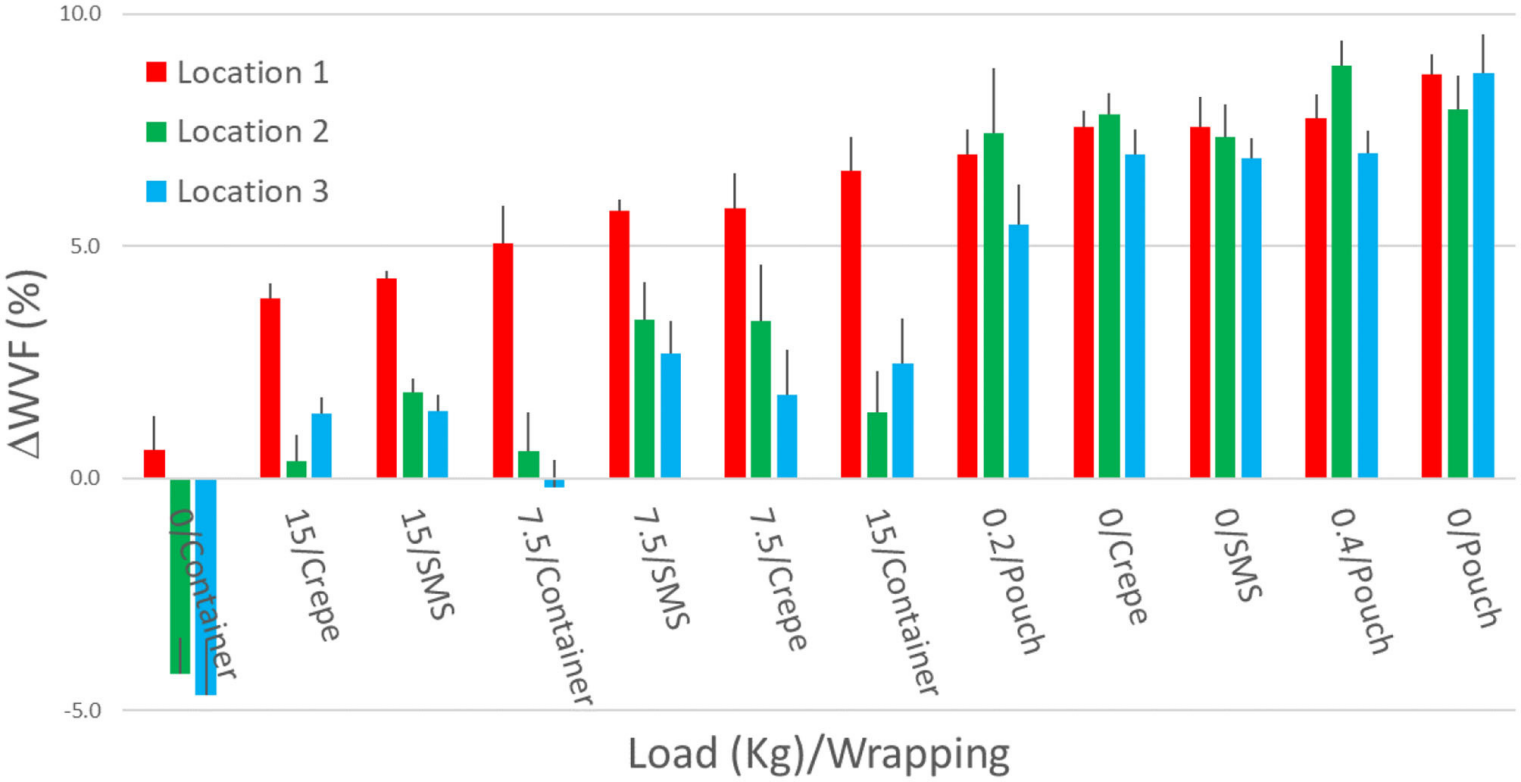
Steam fraction differences (ΔWVF) between the test and reference tubes at the beginning of the exposure phase in different experimental conditions. Values are presented as means and standard error over five replicates. Colors indicate the three locations of the packs inside the sterilizer chamber. Positive ΔWVF values indicate higher steam penetration in the test tubes than in the reference tube (i.e., steam penetration is worse in the reference than test tubes).

ΔWVF values calculated at the beginning of the exposure phase for the different load/wrapping combinations are summarized in Table 2 and shown in Figure 4. Differences ranged from −5 to +9% and were associated to the load/wrapping combination and pack position into the sterilizer chamber.

**Table 2:** WVF differences (ΔWVF) between the test and reference tube sensors, calculated at the beginning of the exposure phase for the different load/wrapping combinations.

**Load/wrapping**	**ΔWVF (%)**		
	**Location 1**	**Location 2**	**Location 3**
0/Crepe	7.6 (0.4)	7.8 (0.5)	7.0 (0.5)
7.5/Crepe	5.8 (0.7)	3.4 (1.2)	1.8 (1.0)
15/Crepe	3.9 (0.3)	0.4 (0.6)	1.4 (0.4)
0/SMS	7.6 (0.6)	7.3 (0.7)	6.9 (0.4)
7.5/SMS	5.8 (0.8)	3.4 (0.8)	2.7 (0.7)
15/SMS	4.3 (0.2)	1.8 (0.3)	1.5 (0.3)
0/Pouch	8.7 (0.4)	8.0 (0.7)	8.7 (0.8)
0.2/Pouch	7.0 (0.5)	7.4 (1.4)	5.5 (0.8)
0.4/Pouch	7.8 (0.5)	8.9 (0.5)	7.0 (0.5)
0/Container	0.6 (0.7)	−4.2 (0.8)	−4.7 (1.2)
7.5/Container	5.1 (0.8)	0.6 (0.8)	−0.2 (0.6)
15/Container	6.6 (0.7)	1.4 (0.9)	2.5 (1.0)

*For each condition data are given as mean (standard error) over five replicates*.

*Location 1, upper front level; location 2, upper rear level; location 3, lower front level*.

*SMS, Spunbond-Meltblown-Spunbond*.

Positive ΔWVF values, testifying that the reference tube was the worst case for steam penetration, were obtained in all the tested combinations, except for the empty container. The latter case displayed a negative ΔWVF value, which indicated poorer steam penetration in the test tubes with open end placed into an empty container than in the reference tube. Since the sterilization of empty containers is not frequent in the practice, the reference tube was considered representative of the worst case in all practical combinations of loads and wrappings usually encountered in hospital settings.

ΔWVF values in Figure 4 pointed out that higher loads were associated with lower steam penetration in soft wrappings (pouch, SMS, crepe), while they were associated with higher steam penetration in rigid systems (i.e., containers).

## Discussion

This work provides for the first time quantitative steam fraction measurements in channels during a typical steam sterilization process for terminal sterilization of reusable medical devices in healthcare settings, with specific focus on the effects of different wrapping material and load amounts on steam penetration. Our experimental results confirmed the initial working hypothesis that a 70 cm long tube could represent the worst case for steam penetration in channels in all practical situations. Indeed, during the sterilization phase, steam penetration in the 70 cm tube was lower than in the wrapped 50 cm long channels, which were representative of the longest channeled instruments and wrapping conditions encountered in the clinical practice.

Our experimental data pointed out that steam penetration was significantly affected by the wrapping material (sterile barrier systems) and the loads present in the pack. Microbiological barrier systems are used to prevent recontamination after the steam sterilization process and are required in terminal sterilization. The wrappings or containing systems used for creating packs may present different steam permeation characteristics related to different materials and designs. Nevertheless, the available evidence on these phenomena is sparse and mostly indirect. A recent experimental study has shown a tendency for increased survivability of bacteria on surgical instruments in wrapped versus unwrapped sets (23). Conversely, another study reported that unwrapped steam sterilization of ophthalmic instruments was coincident with higher rates of postoperative infections (24), although the result was not statistically significant. Of note, relevant standards (25, 26) indicate how to test air permeance through microbiological barrier systems, but do not specify any requirement for steam penetration. In this study we applied a different perspective and evaluated the effect of the wrapping system on steam penetration into channels in a direct and systematic way, providing quantitative measurements of WVF. We showed that empty containers represented the most critical condition for steam penetration among all tested load/wrapping combinations, being characterized by poorer steam penetration with respect to the 70 cm tube. It is worth to notice that empty containers are not commonly encountered in the sterilization practice, and thus the 70 cm metallic tube can be considered representative of the worst case for steam penetration in all practical situations. Interestingly, our results pointed out differences between rigid and soft wrappings at changing loads occur. Heavier loads were associated with higher steam penetration in rigid packaging systems. This finding may be related to the increased duration of the sterilization process (longer pre-conditioning phase) for heavier loads and are consistent with previous studies that have shown higher loads to be more easily penetrated by steam (27, 28). Instead, lighter loads require shorter conditioning times, limiting steam penetration associated with diffusion (28). The amount of load in the pack can also impact steam penetration. Indeed, the condensate and steam flux in the pack is proportional to the mass and specific heat capacity of the load, where a higher mass and specific heat capacity require larger steam volume for load warming. Of note, we observed a different behavior in soft wrappings, such as SMS, pouches, crepe, where a lower steam penetration occurred in the presence of heavier loads. The difference between containers and soft wrappings could be partially related to differences in steam permeation through different microbiological barriers, but also to differences between soft and rigid packs during the conditioning phase. Major changes in chamber pressure can induce changes in the volume of soft packs, but not in rigid containers, which may modify steam availability and gas mixture dynamics inside the packs.

It is worth to notice that our findings are likely extendable to channeled instruments with different diameters from those used in this study. Indeed, variations in the tube internal diameter from 0.7 to 5 mm were shown not to affect WVF measurements at the start of the exposure phase (13). This diameter range is representative for the majority of channeled medical devices for minimally invasive surgery.

Finally, the results of this study may be applicable to both thin and thick-walled channeled instruments. In thin-walled instruments (i.e., channels presenting a warming time of the inner wall equal or shorter than the time required for steam penetration into the channel) channels are mainly warmed though steam condensation on the outer surface (29), while in thick-walled instruments warming occurs by steam condensation on the inner channel wall, creating a non-negligible amount of condensate inside the channel. For this reason, to avoid channel obstruction by water droplets and impaired steam penetration, the orientation of thick-walled instruments during steam sterilization should guarantee an effective condensate drainage from the open end, in order. Provided that the correct orientation of thick-walled channels in the sterilizer is guaranteed (29), there is currently no available indication that steam penetration in thick-walled channels should be more difficult than in thin-walled ones.

### Limitations of the Study

The present study had some limitations. First, steam fraction values measured by the 4D IR sensors do not represent absolute steam fraction values, but only relative measurements. Although the sensors were calibrated prior to use, no independent measurement of steam fraction was available for comparison during this study. Nevertheless, the comparative design of the study, which focused on steam fraction differences rather than absolute values, granted the robustness of our findings.

An additional limitation, not compensated by the study design, was related to the potential influence of the tube material on experimental measurements. Due to experimental constrains, the reference tube was made of stainless-steel, while the test tubes were realized with flexible silicone and PTFE. Differences in tube materials may have partially influenced steam fraction measurements. Nevertheless, previous studies have shown that in thin-walled channels changes in heat capacity and conductivity of the tube material do not significantly affect steam penetration into the channel, provided that the outer surface of the tube was immediately warmed by direct steam exposure and that heat transfer from the outer to the inner surface occurred within seconds (11). These assumptions should be satisfied by the tubes used in this study, although small deviations may occur for silicone and PTFE tubes as it has been previously postulated by Borchers and Mielke in experiments with microbial spores (16). Finally, the absorption and release of water molecules by silicone may influence steam fraction measurements acting as a secondary water source in the tube (12, 16). Although this effect is expected to be secondary, it would be valuable to perform further studies using 50 cm stainless-steel tubes in the packs.

## Conclusion

The results of this experimental study, designed to test a wide variety of load, loading patterns, and sterile barriers systems typical of in-field applications, provided evidence that a 70 cm long tube with one end open and one end closed is representative of the worst case for steam penetration in channeled instruments in practical situations. This means that reaching a sufficient steam penetration into such a designed reference tube guarantees an adequate penetration in the channeled instruments to be sterilized, irrespective of the wrapping, position, and additional load into the pack. Thus, a steam fraction sensor equipped with this tube may constitute an alternative, physics-based and objective steam penetration test to monitor in real time the processing of massive and channeled instruments. These unique features may favor the development of an automatized and reliable monitoring system for steam penetration into channeled instruments and potentially promote a more efficient management of the sterilization process.

## Data Availability Statement

The raw data supporting the conclusions of this article will be made available by the authors, without undue reservation.

## Author Contributions

FT and JD: conceptualization, formal analysis, resources, supervision, and project administration. FT, MM, and AV: methodology. FT, MM, AV, and JD: investigation. MM and AV: data curation. FT and MM: writing—original draft preparation. JD and AV: writing—review and editing. FT: funding acquisition. All authors: contributed to the article and approved the submitted version.

## Conflict of Interest

JD was employed by the company Steelco s.p.a, Italy. The remaining authors declare that the research was conducted in the absence of any commercial or financial relationships that could be construed as a potential conflict of interest.
